# A comparative study between CT, MRI, and intraoral US for the evaluation of the depth of invasion in early stage (T1/T2) tongue squamous cell carcinoma

**DOI:** 10.1007/s11282-021-00533-7

**Published:** 2021-05-10

**Authors:** Masaki Takamura, Taichi Kobayashi, Yutaka Nikkuni, Kouji Katsura, Manabu Yamazaki, Satoshi Maruyama, Jun-ichi Tanuma, Takafumi Hayashi

**Affiliations:** 1 Division of Oral and Maxillofacial Radiology, Niigata University Graduate School of Medical and Dental Sciences, 2-5274 Gakkocho-dori, Chuo-ku, Niigata, 951-8514 Japan; 2 Division of Oral Pathology, Niigata University Graduate School of Medical and Dental Sciences, 2-5274 Gakkocho-dori, Chuo-ku, Niigata, 951-8514 Japan; 3 Oral Pathology Section, Department of Surgical Pathology, Niigata University Medical & Dental Hospital, 1-754 Asahimachi-dori, Chuo-ku, Niigata, 951-8520 Japan

**Keywords:** Tongue carcinoma, DOI, Intraoral sonography, CT, MRI

## Abstract

**Objectives:**

This study aimed to clarify the accuracy of intraoral ultrasonography (US), computed tomography (CT), and magnetic resonance imaging (MRI) in preoperative image depth of invasion (DOI) measurement of T1/T2 tongue cancer through comparison with histopathological measurements.

**Methods:**

Imaging of the primary lesions was performed at our hospital; the lesions were classified into T1 and T2 based on the 8th edition of the AJCC/UICC, and surgery performed. There was histopathological confirmation of lesions as squamous cell carcinoma in 48 patients with tongue cancer. T3 and T4 cases, cases in which preoperative chemotherapy and radiation therapy were performed, and cases where biopsy was performed before imaging were excluded. The radiological DOI in US, CT, and MRI and the histopathological DOI as base were comparatively investigated and statistical analyses were performed by Bland–Altman analysis and Spearman's rank correlation coefficient.

**Results:**

Bland–Altman analysis showed that the US radiological DOI was overestimated by an average of 0.2 mm compared to the histopathological DOI, while CT and MRI radiological DOI were overestimated by an average of 2–3 mm. The comparison of CT and MRI revealed that the difference between the MRI and histopathological DOI, as well as the 95% limit of agreement, were smaller than those of the CT radiological DOI.

**Conclusions:**

US is the most accurate preoperative diagnostic tool for T1 and T2 squamous cell carcinoma; CT and MRI tend to have an overestimation of about 2–3 mm and so caution is required.

## Introduction

Tongue cancer is the most frequent among oral cancers [1] and highly effective diagnosis and treatment is extremely important. In particular, it is known that the deeper the invasion of the primary lesion, the higher the frequency of metastases to the cervical regional lymph nodes, which is associated with a poor prognosis [2–6]. In response to this phenomenon, in 2017, the 8th edition of the TNM classification of the Union Internationale Contre le Cancer (UICC) and the 8th edition of the American Joint Committee on Cancer (AJCC) introduced the concept of depth of invasion (DOI) in addition to the superficial spread of the tumor to the T classification. DOI is defined histopathologically as the vertical distance from the virtual plane connecting the basement membrane of the normal mucosa adjacent to the tumor to the deepest part of the tumor and is different from the thickness of the tumor. DOI is roughly divided into three categories: (1) 5 mm or less, (2) more than 5 mm but no more than 10 mm, (3) more than 10 mm and in the case of tongue cancer with DOI exceeding 5 mm, preventive neck dissection and follow-up after treatment is to be considered [7, 8]. However, clinical measurement methods, including diagnostic imaging, have not been clearly defined and have not yet been formulated. Although the 8th edition of UICC/AJCC states that CT or MRI is effective for preoperative examination [9], the standard method of preoperative DOI measurement has not yet been established.

In recent years, ultrasonographic diagnosis has come to be widely used as a diagnostic imaging of the head and neck region. Although the UICC and AJCC state that ultrasonography is not suitable for the evaluation of primary lesions [9], some studies have evaluated primary lesions with intraoral ultrasonography (US) and have shown a strong correlation with histopathological thickness or DOI [10–13]. To date, several reports have been made on CT, MRI, and US preoperative radiological DOI evaluations, but there is no report of a study where these were all performed in a single institution [10–28]. Therefore, this study was aimed to investigate the accuracy of preoperative radiological DOI measurement with CT, MRI, and US in the same target group based on histopathologically measured DOI for T1 and T2 tongue cancers, where a diagnosis of DOI around 5 mm is an important diagnostic finding and examine the effectiveness of each imaging modalities with retrospective approach.

## Materials and methods

### Patients

The subjects of this retrospective study were patients with a primary lesion of the tongue confirmed by oral and maxillofacial radiologists in our hospital. They underwent CT, MRI and US imaging modalities followed by surgery after the lesions were classified into T1 and T2 based on the 8th AJCC/UICC edition between April 2014 and March 2019. There were 48 cases of tongue cancers histopathologically confirmed as squamous cell carcinoma. Cases with stage T3–T4 and those treated with neoadjuvant chemotherapy or radiation therapy were excluded.

This study was approved by our institutional review board (No. 2019-0219) and informed consent was obtained from all participants. The guidelines of the Helsinki Declaration were followed during this investigation.

### Imaging protocol

CT was performed with a 64-row multi-detector row CT (Ingenuity Elite; Philips Medical Systems, Best, Netherlands) or 320-row area-detector row CT (Aquilion ONE, CANON Medical Systems, Tokyo, Japan). The imaging range was from the inferior margin of the orbit to the superior mediastinum. Intravenous administration of contrast medium was performed in all cases. The contrast medium was injected from superficial veins of the cubital fossa or forearm at a speed of 0.8–1.5 mL/s using a nonionic iodine contrast medium with an iodine content of 300–350 mg/mL, and the imaging was started 70 s or 90 s after the contrast medium was injected. For all devices, the tube voltage was 120 kV and the tube current was automatically adjusted, with a slice thickness of 1 mm and a field of view (FOV) of 220 mm were set, and the Pitch Factor was 0.391 for Ingenuity Elite and 0.637 for Aquilion ONE. A coronal reconstruction image (MPR) with a 2 mm interval was created from the axial image.

MRI was performed with a 1.5-T MRI (EXCELART Vantage Titan, CANON Medical Systems, Tokyo, Japan) or 3.0-T MRI (Discovery MR750w, GE Healthcare, Milwaukee, US). A gadolinium preparation was the contrast agent used that was injected from superficial veins of the cubital fossa or forearm in all cases. Fat suppressed T2-weighted images (FST2-WI) of the axial and coronal sections and fat suppressed post-contrast T1-weighted images (FSCET1-WI) of the axial and coronal sections were captured. The FST2-WI of the 1.5-T MRI was set at TR/TE, 4200 ms/84 ms; flip angle, 90°; FOV, 230 mm; matrix size, 512 × 512; in the axial section, slice thickness was 5 mm and slice gap, 0 mm; and in the coronal section, slice thickness was 5 mm and slice gap, 1 mm. The FST2-WI was set at TR/TE, 500 ms/10 ms; flip angle, 90°; FOV, 230 mm; matrix size, 512 × 512, in the axial section, slice thickness was 5 mm and slice gap, 0 mm; and in the coronal section, slice thickness was 5 mm and slice gap, 1 mm. For the 3.0-T MRI FST2-WI, the FOV was set at 230 mm and matrix size, 512 × 512, for the axial section, TR/TE was set at 4054 ms/89.14 ms; slice thickness, 5 mm and slice gap, 1 mm; for the coronal section, TR/TE was set at 3195 ms/81.47 ms; slice thickness, 4 mm and slice gap, 1 mm. For the FSCET1-WI, the FOV was 230 mm and matrix size, 512 × 512, for the axial section, TR/TE was, 622 ms/9.31 ms; slice thickness, 5 mm and slice gap, 1 mm; for the coronal section, TR/TE was 558 ms/9.28 ms, slice thickness, 4 mm and slice gap, 1 mm.

US was performed with a stationary ultrasound diagnostic device HI VISION Preirus (Hitachi, Tokyo, Japan) and a hockey stick-type intraoperative small transducer (EUP-O54J) with a frequency of 7 to 13 MHz, to perform oral scanning between the tumor and the transducer via a polymer acoustic coupling material (SONAGEL; Takiron, Osaka, Japan or Echo Gel PAD; Yasojima Proceed, Hyogo, Japan) and food wrap for infection and contamination prevention. Imaging was performed in B mode using tissue harmonics and spatial compounds, the tongues of patients were lightly held with gauze, and scanning was performed in a cross section close to the axial section, with minimal pressure in order to minimize deformation of the cancer tissue and patient discomfort. The image was confirmed by real-time video, the tumor depicted in the low echo compared with the surrounding muscle tissue was stilled in the thickest and clearest cross section, and the image was preserved. The scope of the tumor was confirmed using the Doppler method (FineFlow^®^) and strain elastography (Real-time tissue elastography). US examinations were performed by four oral and maxillofacial radiologists familiar with intraoral ultrasound scanning.

### Evaluation of radiological DOI

For CT, MRI, and US, radiological DOI was measured on a dedicated terminal from DICOM data transferred and stored in PACS (Synapse Viewer; Fuji Medical Systems, Tokyo, Japan).

In CT, the measurement was performed using the post-contrast axial image and the post-contrast coronal MPR image. The display was soft tissue display, and the window width and window value were set to 300/40 or 350/30. Measurements by MRI were performed the axial and coronal images of FSCET1-WI and FST2-WI. With US, measurement were performed using the most appropriate images with the clearest depiction of the tumor from among the captured images taken by four oral and maxillofacial radiologists.

**Fig. 1:**
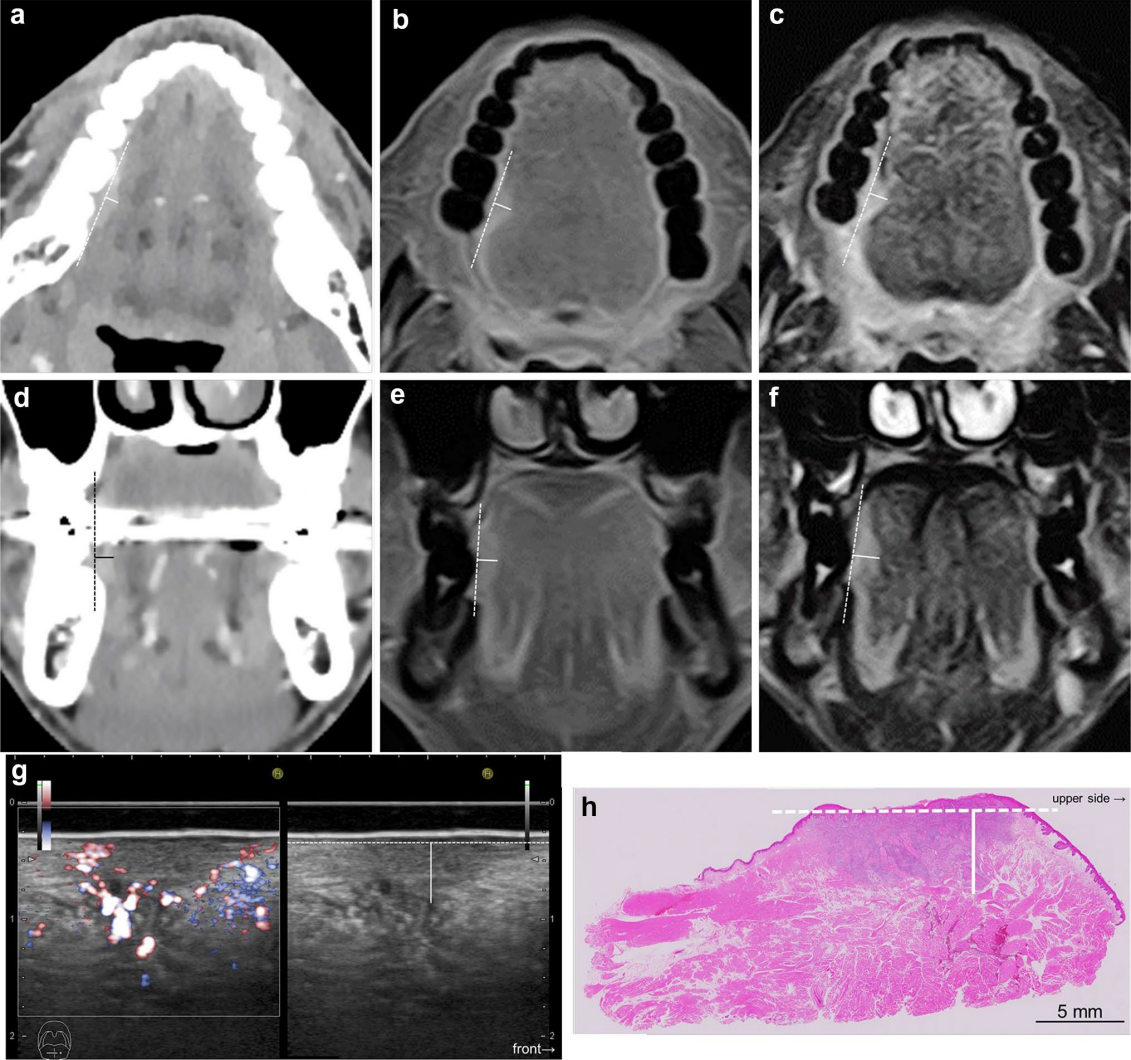
Measurement of radiological depth of invasion (DOI) and histopathological DOI in squamous cell carcinoma of the right lateral tongue. Axial post-contrast computed tomography (CT) **a**; DOI = 4 mm, axial fat-suppressed post-contrast T1-weighted images (FSCET1-WI) **b**; DOI = 5 mm, axial fat-suppressed T2-weighted images (FST2-WI) **c**; DOI = 5 mm, coronal post contrast MPR-CT **d**; DOI = 3 mm, coronal FSCET1-WI e; DOI = 6 mm, coronal FST2-WI **f**; DOI = 7 mm, intraoral ultrasonography (US) (left: Doppler method, right: B mode image) **g**; DOI = 4 mm, pathology **h**—DOI = 5 mm. For radiological DOI in CT and MRI, the vertical distance (solid line) from the virtual line (dotted line) connected the boundary between the tumor and the normal mucosa to the deepest part of the tumor. US showed a vertical distance (solid line) from the virtual line (dotted line) connecting the base of the normal mucosal portion adjacent to the tumor to the deepest part of the tumor (solid line). If the entire lesion was not visible due to metal artifacts, it was measured within an observable range. For the histopathological DOI, the vertical distance (solid line) from the virtual line (dotted line) connecting the normal mucosal basement membrane adjacent to the tumor to the deepest part of the tumor was measured histopathologically

**Fig. 2:**
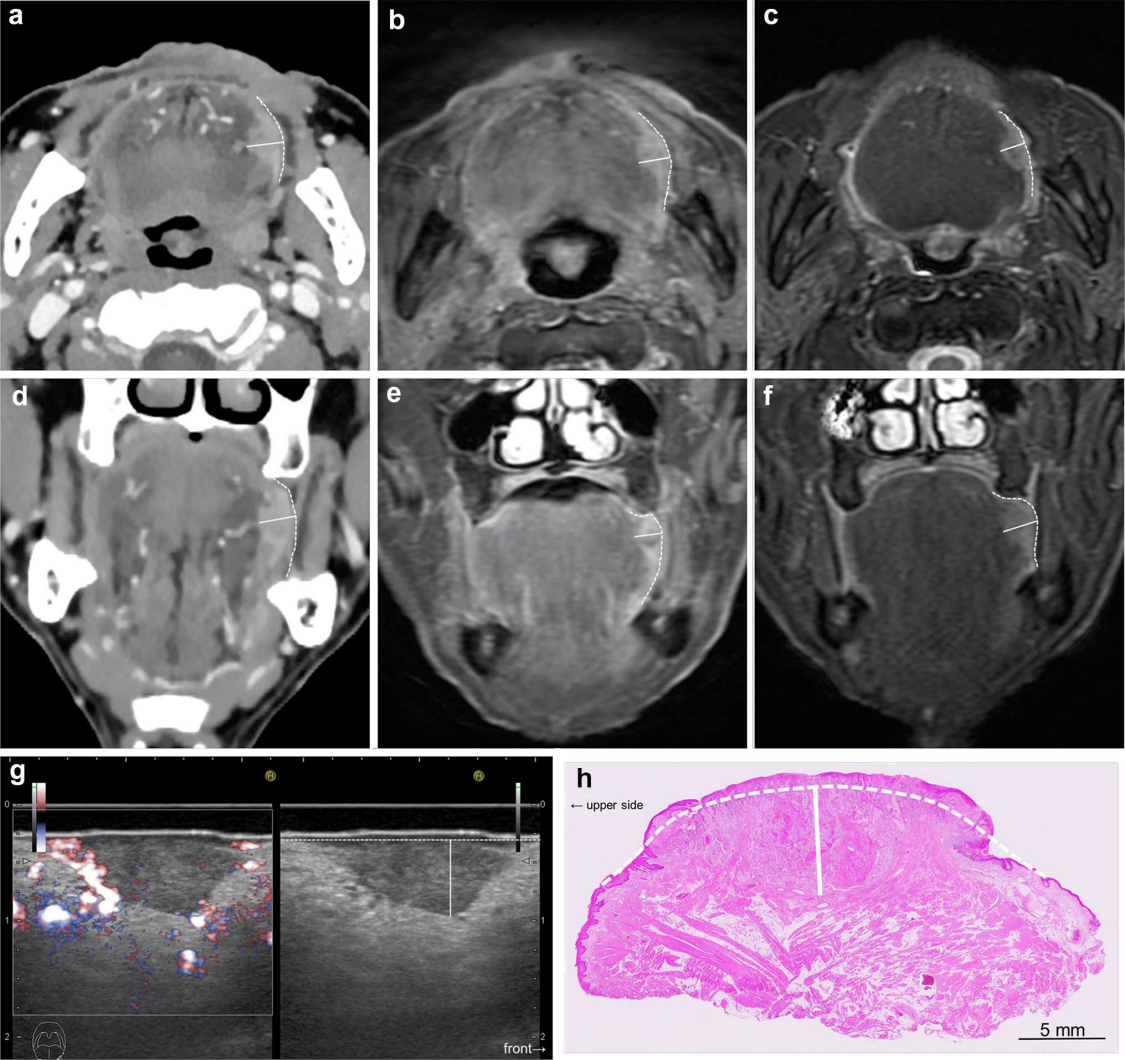
Measurement of radiological DOI and histopathological DOI in squamous cell carcinoma of the left lateral tongue. Axial CT **a**; DOI = 9 mm, axial FSCET1-WI **b**; DOI = 6 mm, axial FST2-WI **c**; DOI = 6 mm, coronal post-contrast MPR-CT **d**; DOI = 9 mm, coronal FSCET1-WI **e**; DOI = 7 mm, coronal FST2-WI **f**; DOI = 7 mm, US (left: Doppler method, right: B mode image) **g**; DOI = 6 mm, pathology **h**—DOI = 6 mm. In CT and MRI, a virtual curve (dotted line) was set with reference to the form of the tongue mucosa on the opposite side and the curve of the normal mucosa, and the perpendicular line (solid line) from the virtual curve to the deepest part of the tumor was taken as the DOI. In the histopathological DOI, a virtual curve (dotted line) was set with reference to the form of the curve of the normal mucosa, and the vertical distance from the virtual curve to the deepest part of the tumor (solid line) was taken as the DOI. The vertical distance from the virtual curve to the deepest part of the tumor (solid line) was taken as the DOI

In this study, radiological DOI was defined as the vertical distance from the virtual line connecting the boundary between the tumor and the normal mucosa to the deepest part of the tumor by CT and MRI. By US, it was the vertical distance from the virtual line connecting the normal mucosal basal portion adjacent to the tumor to the deepest part of the tumor. All modality images were evaluated visually, and the image where the deepest part of the tumor was depicted, and its location were determined by consensus. For CT and MRI, when the virtual line was judged inappropriate for a straight line depending on the location and size of the tumor, the virtual curve was set in reference to the curvature of the normal mucosa or the tongue on the opposite side, and the measurement was carried out within the observable range. (Figs. 1, 2) Measurement of the radiological DOI was performed by two oral and maxillofacial radiologists (supervisory oral and maxillofacial radiologists, one with 33 years of experience and the other with 3 years of experience), and the measurement value was determined by consensus to avoid the interobserver variability.

### Evaluation of histopathological DOI

The excised specimen was fixed with 10% formalin solution, and hematoxylin eosin staining was performed. In the eighth edition of the AJCC/UICC, DOI is defined as the vertical distance from the virtual line connecting the basement membrane of the normal mucosa adjacent to the tumor to the deepest part of the tumor. Therefore, measurements were performed by an oral pathologist according to this definition. When the virtual line was judged inappropriate as a straight line, a virtual curve was set and measured in reference to the curvature of the normal mucosa. This measurement value was used as the histopathological DOI.

### Statistical analysis

With the histopathological DOI as standard, comparison with the radiological DOI was performed by Bland–Altman analysis. In the Bland–Altman analysis, the mean values of the histopathological DOI and radiological DOI were plotted on the X-axis and the difference between the histopathological DOI and radiological DOI on the Y-axis [29, 30]. In addition, the correlation between the histopathological DOI and radiological DOI was performed by Spearman’s rank correlation coefficient. A *p*-value below 0.05 indicated a statistically significant difference. All statistical analyses were performed by SPSS (IBM Japan Inc., Tokyo, Japan).

## Results

Of the 48 subject patients, there were 28 men and 20 women, with a mean age of 65.7 years (23–90 years). There were 28 T1 cases and 20 T2 cases. Histopathologically, 40 cases had a DOI of 5 mm or less, and 8 cases of more than 5 mm but below 10 mm with a mean value of 3.3 mm. There were 26 cases classified as T1N0, 17 as T1N1, 2 as T2N0, and 3 as T2N1. Clinically, the greatest diameter of the tumor was between 5 and 39 mm with a mean of 20.0 mm. Resection of the primary lesion and neck dissection were performed simultaneously in five patients, and histopathological lymph node metastasis was observed in three patients. Of the 43 cases where only the primary lesion was resected, there was a recurrence of cervical lymph node metastasis in 8. No patient had local recurrence. When a lesion could not be identified via CT and MRI due to metal artifacts, or biopsy was performed before the study, such patients were excluded from the study. Twenty-seven patients underwent CT; 25 cases were performed by Ingenuity Elite and two cases were performed by Aquilion ONE. Fifteen patients underwent post-contrast fat-suppressed T1-weighted imaging (FSCET1-WI); 11 cases were performed by 1.5 T MRI and four cases were performed by 3.0 T MRI. Sixteen patients underwent fat-suppressed T2-weighted imaging (FST2-WI); 12 cases were performed by 1.5 T MRI and four cases were performed by 3.0 T MRI. Thirty-eight patients underwent US. The mean number of days from imaging to resection was 16.8 days (8–34 days) for MRI, 24.6 days (2–50 days) for CT, and 22.4 days (1–58 days) for US. Tumors were located in lateral tongue for 43 cases, in inferior tongue for three cases, in tongue tip for two cases.

Histopathologically, the mean DOI was 3.3 mm (0–10 mm). For the radiological DOI measured by CT, the mean measurement of the axial section was 6.1 mm (2–11 mm), and that of the coronal section was 6.1 mm (2–10 mm). For radiological DOI measured by MRI, the mean measurement of the FSCET1-WI axial section was 6.1 mm (2–12 mm); that of the coronal section was 6.3 mm (2–10 mm). The mean measurement for the FST2-WI axial section was 6.1 mm (2–13 mm); that for the coronal section was 6.6 mm (3–12 mm). In the radiological DOI measured by US, the mean value was 3.6 mm (0–9 mm) (Table 1).

**Table 1:** Summary of statistical analysis on Bland–Altman plot and Spearman’s rank correlation coefficient

	Cases	Mean of rDOI	Mean of pDOI	Bias	SD	95% limit of agreement	Spearman's *r*s
Intraoral US	38	3.6	3.4	0.2	1.4	– 2.6 to 2.9	0.83
CT (axial section)	27	6.1	3.5	2.6	2.4	– 2.1 to 7.4	0.62
CT (coronal section)	27	6.1	3.5	2.6	2.5	– 2.4 to 7.5	0.58
MRI (CET1WI, axial section)	15	6.1	4.2	1.9	1.9	– 1.7 to 5.6	0.73
MRI (CET1WI, coronal section)	15	6.3	4.2	2.1	1.6	– 1.1 to 5.2	0.79
MRI (T2WI, axial section)	16	6.1	4.1	2.0	2.1	– 2.1 to 6.1	0.66
MRI (T2WI, coronal section)	16	6.6	4.1	2.5	1.5	– 0.4 to 5.4	0.83

**Fig. 3:**
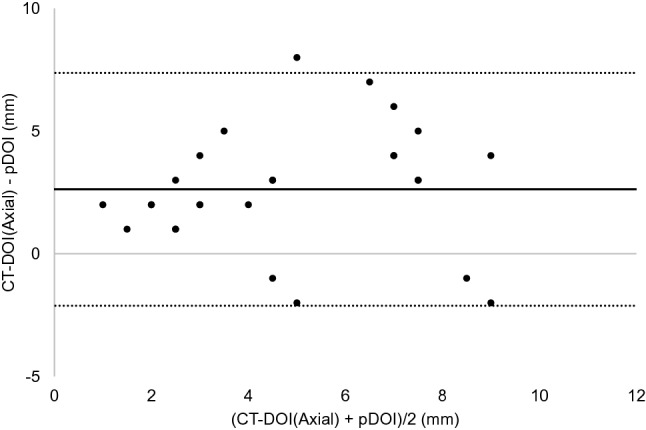
Bland–Altman plot of radiological DOI (rDOI, CT, Axial section) and histopathological DOI (pDOI). Mean difference between rDOI and pDOI is 2.6 mm, 95% limits of agreement is − 2.1 to 7.4 mm

**Fig. 4:**
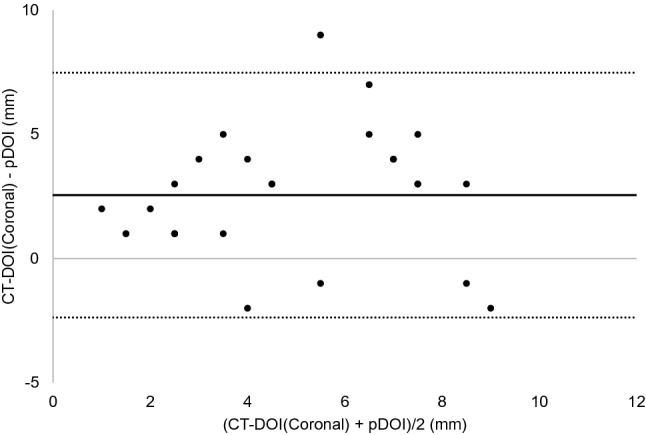
Bland–Altman plot of rDOI (CT, Coronal section) and pDOI. Mean difference between rDOI and pDOI is 2.6 mm, 95% limits of agreement is − 2.4 to 7.5 mm

**Fig. 5:**
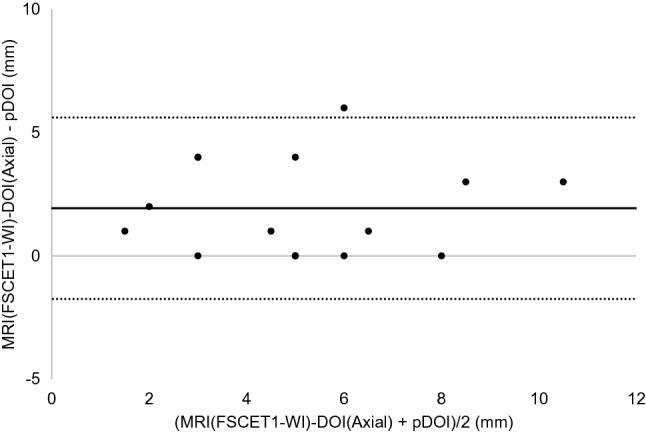
Bland–Altman plot of rDOI (FSCET1-WI MRI, Axial section) and pDOI. Mean difference between rDOI and pDOI is 1.9 mm, 95% limits of agreement is − 1.7 to 5.6 mm

**Fig. 6:**
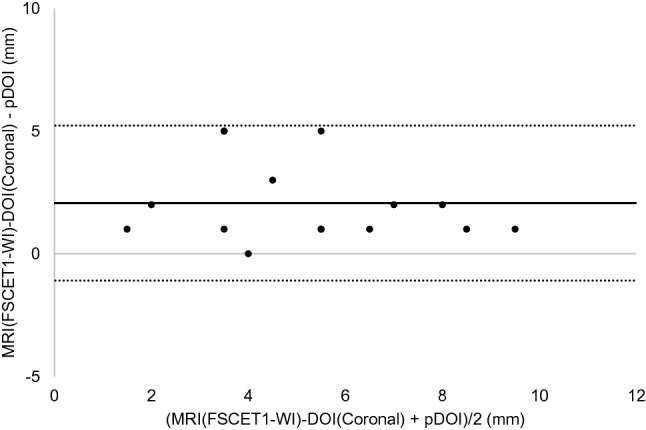
Bland–Altman plot rDOI (FSCET1-WI MRI, Coronal section) and pDOI. Mean difference between rDOI and pDOI is 2.1 mm, 95% limits of agreement is − 1.1 to 5.2 mm

**Fig. 7:**
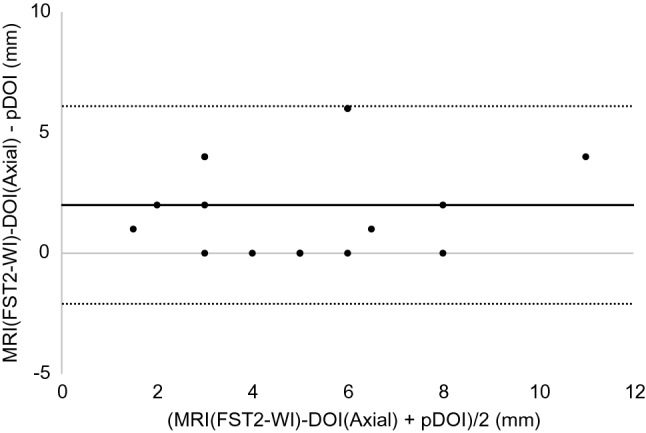
Bland–Altman plot of rDOI (FST2-WI MRI, Axial section) and pDOI. Mean difference between rDOI and pDOI is 2.0 mm, 95% limits of agreement is − 2.1 to 6.1 mm

**Fig. 8:**
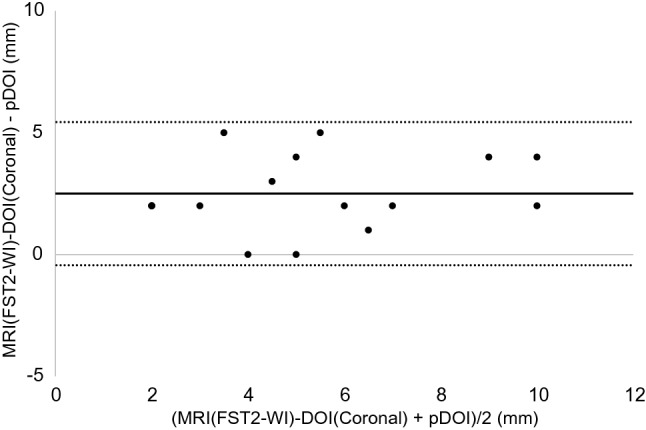
Bland–Altman plot of rDOI (FST2-WI MRI, Coronal section) and pDOI. Mean difference between rDOI and pDOI is 2.5 mm, 95% limits of agreement is − 0.4 to 5.4 mm

**Fig. 9:**
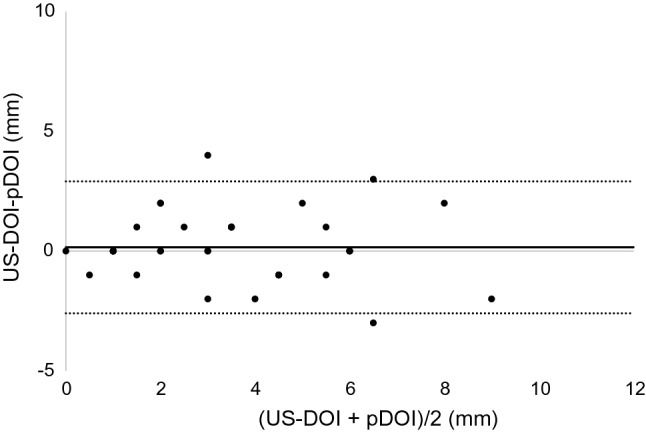
Bland–Altman plot of rDOI (intraoral US) and pDOI. Mean difference between rDOI and pDOI is 0.2 mm, 95% limits of agreement is − 2.6 to 2.9 mm

The results of the Bland–Altman analysis were as shown below. For CT, the difference (bias) between the histopathological DOI and CT radiological DOI was 2.7 mm with the axial section, 2.6 mm with the coronal section, and the 95% limit of agreement were − 2.1 to 7.4, − 2.4 to 7.5 mm (Figs. 3, 4). With the MRI radiological DOI, bias in the FSCET1-WI axial section and coronal section; bias in the FST2-WI axial section and coronal section was 1.9, 2.1, 2.0, and 2.5 mm, respectively, and the 95% limit of agreement were − 1.7 to 5.6, − 1.1 to 5.2, − 2.1 to 6.1, and − 0.4 to 5.4 mm, respectively (Figs. 5,6,7, and 8). With the US radiological DOI, the bias was 0.2 mm and the 95% limit of agreement was − 2.6 to 2.9 mm (Fig. 9) (Table 1.). Comparison of CT and MRI showed that the bias and 95% limit of agreement were slightly greater in CT. Comparison of T1-WI and T2-WI in MRI showed that T2-WI overestimated lesions more.

Spearman's rank correlation coefficient, rs, was 0.62 for axial CT, 0.58 for coronal CT, 0.73 for axial FSCET1-WI, and 0.79 for the coronal sections, 0.66 for axial FST2-WI, and 0.83 for the coronal sections. For US, it was 0.83 (*p* < 0.05) (Table 1).

## Discussion

DOI was introduced in the UICC/AJCC 8th edition of the T classification of oral cancers, including tongue cancer that was published in 2017. DOI is defined histopathologically as the vertical distance from the virtual plane connecting the basement membrane of the normal mucosa adjacent to the tumor to the deepest part of the tumor, and is a factor associated with survival prognosis, including the risk of metastasis to the cervical lymph nodes, reported in many papers [2–6]. If DOI can be measured during preoperative diagnostic imaging, it would be useful for determining treatment policies and predicting prognosis, but no specific method of DOI measurement in diagnostic imaging has been shown [9]. Therefore, in this study, the accuracy of diagnostic imaging was estimated by comparing the DOI measured by CT, MRI, and US with the DOI measured histopathologically in the same target group. Few studies have evaluated primary lesions of tongue cancer by CT [14, 15, 31]. Although CT has better spatial resolution than MRI, metal artifacts often make it difficult to evaluate lesions. The oral cavity has many metal restorations, making it difficult to assess primary lesions in many cases. Of the 38 patients that were examined by CT scan in this study, the lesion could not be clearly identified in 11. Although 27 patients were evaluable, there may have been a mixture of patients where appropriate imaging of the cross section could not be performed due to metal artifacts and the actual depth of the tumor was not captured. This was likely one of the factors that made the 95% limit of agreement in the Bland–Altman analysis larger than that of MRI. Previous reports have suggested that CT is accurate enough to assess the primary lesion of tongue cancer [14, 15, 31], and this effect should also be taken into consideration.

Since MRI is less susceptible to metal artifacts, it is expected to be superior to CT for DOI measurement. The accuracy varies across reports, with some suggesting it was almost consistent with the histopathological DOI [26] or overestimated by about 2 mm [21, 24, 28]. In this study, an overestimation of about 2–3 mm was observed overall. It should be noted that the overestimation was caused by peritumoral edema and reactive inflammation [16, 25, 32]. Such histopathological changes of the surrounding area may have affected these results also, indicating that not only the tumor per se is detected. Such effects seem to occur similarly even in CT. However, there might be enough data to refer to the influence of partial volume effects based on differences with MRI regarding slice thickness and voxel size.

For US, Bland–Altman analysis suggested that the bias and the width of the 95% limit of agreement was smaller than that of CT and MRI, suggesting that the extent of tumor invasion was more likely to have been captured almost accurately. A small hockey stick-type intraoperative transducer is used as a scanning method for US and perform oral scanning between the tumor and the transducer via a polymer acoustic coupling material. Although there are restrictions due to the examination being performed in a narrow oral cavity and in some cases involves considerable contact pain, it is possible to measure the tumor by applying the transducer from any direction to some extent. In addition, similar to CT and MRI, it seems that US was affected by edema and reactive inflammation of the peritumoral tissue. However, with post-contrast CT and post-contrast MRI, the tissue enhanced by a contrast agent was depicted, whereas, US seems to have detected a range closer to the tumor body as a hypoechoic lesion based on the difference in acoustic impedance with the surrounding tissue. Here, CT and MRI showed an overestimation of 2–3 mm, while the overestimation of 0.2 mm in the US was almost consistent with the histopathological DOI. It is therefore possible that US is not affected by the edema and inflammation of the peritumoral tissue as much as CT and MRI. However, in some cases, the examination is not possible if the patient has a mouth opening limitation or if the cancer has developed near the root of the tongue where the transducer does not reach, or if the contact pain is obvious. Although the tumor location of most cases (43 of 48) were lateral portion of the tongue and therefore, intraoral scanning was performed without marked difficulty in this investigation, it is judged that other modalities should be prioritized in such situations. There have also been reports of decreased accuracy in cases with histopathological DOI greater than 5 mm [11, 12], and caution should be paid when using US in advanced cancer cases. In this study, T3 and T4 cases were excluded, and as there were only eight cases where the histopathological DOI exceeded 5 mm, such a tendency was rarely observed. Given that there is no concern about exposure to ionizing radiation, for patients that cannot undergo contrast imaging tests due to renal disorders, allergies etc., US is a non-invasive and inexpensive test compared to CT and MRI. In addition, there are reports stating that lesions with a DOI of 5 mm or less could hardly be identified by CT and MRI [23, 33]. US should be used proactively for lesions with shallow depth of invasion, and it is reasonable to make US the first choice for preoperative diagnostic imaging of early tongue cancer. In addition, US has some disadvantages, such as the lack of objectivity in images and the dependence of the accuracy on the examiner, which can be ensured to some extent by the rationalization of the evaluation criteria and organizational training system.

**Fig. 10:**
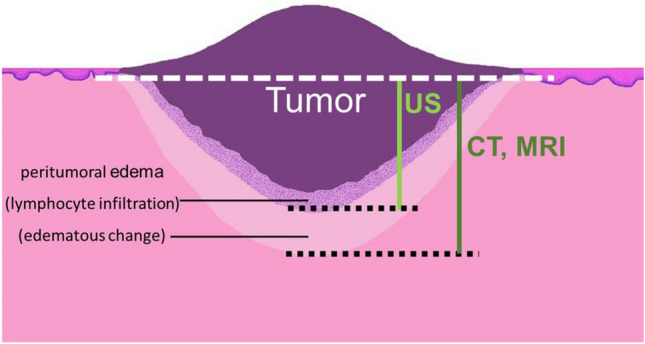
Conceptual schematic drawing of the peritumoral edema in the normal mucosal tissue around the tumor (cancer). Note the difference of the deepest portion of tumor invasion between CT/MRI and US. The interpretations of the tumor on CT and MRI may include the edematous change of the surrounding tissue

Overestimation of lesions was observed in all modalities in this study as mentioned above. Previous reports have described edema and reactive inflammation of peritumoral tissue as factors of overestimation in MRI, but as we have confirmed through this study, the extent of inflammatory cell infiltration in peritumoral tissue that could be observed histopathologically was very limited, not as much as 2–3 mm. In other words, it was thought that edematous changes associated with circulatory disorders in surrounding tissues, which are not clearly reflected by histopathology, accounted for most of the factors of overestimation in MRI. For CT and MRI, an intravenously administered contrast agent reaches its maximum concentration in the circulating blood in 1–2 min; first, the dilated vascular cavity and the increased vascular bed are enhanced [34–36], then the contrast agent is transferred to the tumor tissue and other tissues according to osmotic pressure. Capillary-rich tumor tissue is contrasted relatively early, but the surrounding tissue with edematous changes has fewer capillaries compared to tumor tissue, and so the contrast agent migrates gradually [34, 35]. Thus, it should be noted that the areas imaged by CT and MRI include not only tumor body, but also the edematous changes portion of the tumor body itself and surrounding tissues. The range captured by each modality can be illustrated as shown in Fig. 10. There have been reports that dynamic MRI captures DOI and thickness more accurately than post-contrast T1-WI [22, 37], which seems to minimize the delineation of edema and reactive inflammation of peritumoral tissue. Dynamic imaging is also being performed at our institution, but there are variations depending on the imaging equipment and conditions. For the same reason, although DWI (diffusion weighted image) is utilized in head and neck region [17], it is not applied in this study. In this study, T1-WI and T2-WI (both of which are fat-suppressed) were applied after imaging, which are more commonly used and can be evaluated stably. When these were compared in this study, the tendency for an overestimation of T2-WI was slightly greater than that of T1-WI. Previous reports have also pointed out the tendency for an overestimation of T2-WI [32]; however, the same is true in this study also. In principle, T2-WI is more sensitive to edematous changes in peritumoral tissue than post-contrast T1-WI and that was likely linked to such a tendency. Since the slice thickness of MRI in this study was 4–5 mm, it may be affected by the partial volume effect in no small way. Studies using a slice thickness of 1–2 mm have been reported [19, 24, 31]; however, as with other reports, an overestimation of about 2 mm has been observed, and the effect of the partial volume effect on the overestimation seems to be less significant. However, it has been reported that with MRI of slice thickness of 4–5 mm, lesions with a DOI of 5 mm and below could hardly be identified [25, 33]. It may therefore be advantageous to reduce the slice thickness for smaller lesions. With regard to CT overestimation, there is little difference in pharmacokinetics between nonionic iodine contrast agents and gadolinium preparations in MRI, although the principles of contrast enhancing are different [34]. The timing of T1-WI performance after imaging is a few minutes after administration of the contrast agent, while that for CT imaging is 70–90 s after intravenous administration of the contrast agent. With this as the timing before the contrast agent migrates to the area of the surrounding tissue with edematous change, the extent of overestimation should be reduced. However, it had the same extent as that of T1-W1 after MRI imaging. This may be due to the effect of the tissue resolution of CT, which is not sufficient. As such, this may need to be examined in detail in the future.

When comparing axial and coronal images in CT and MRI, for both post-contrast T1-WI and T2-WI in MRI, the Bland–Altman analysis revealed that the 95% limit of agreement was small for the coronal sections. This may be because the cutting out of the excised specimen is performed in the cross section close to the coronal section. CT, on the other hand, showed no significant difference between the axial and coronal sections (MPR images). Previous CT studies have shown better results for lingual lesions when axial sections were compared with coronal and sagittal sections [18]. This may also be influenced by the limitations of CT tissue resolution.

When comparing two devices about CT and MRI, 25 cases were performed by Ingenuity Elite (64-row multi-detector CT) and two cases were performed by Aquilion ONE (320-row area detector CT). In MRI, 12 cases were performed by 1.5 T MRI, four cases were performed by 3.0 T MRI. There are not enough data supporting the influence between two different CT scanners, also between two different MRI machines.

For histopathological tissues, it is known that the excised tissue shrinks in the process of specimen production. The extent of these effects varies from report to report, but it is said to be between 20.2 and 34.7% [9, 38–40]. Although this study is based on histopathological specimens, the value seems to be about 20–30% smaller than the DOI in the actual living organism. However, on the other hand, it takes about 2–3 weeks on average from the examination by each modality to the surgery, and it is very likely that the tumor has increased in no small way during this time. In this study, the US results were very accurate, while CT and MRI results were overestimated by about 2–3 mm. This seems to have been derived from the intertwining of such complex factors; however, it does not go beyond speculation. The accuracy of the US shown in this study may actually be to some extent underestimated. To eliminate these effects and achieve more accuracy, it is necessary to conduct an imaging examination again before surgery. However, this is not practical as its purpose is not stage classification, which helps in determining the treatment plan. Basically, in the US procedure, a patient's tongue is slightly pulled by the examiner, and a transducer applied to the tumor is lightly pressed for scanning. Although polymer acoustic coupling materials act as buffers, tumors may be compressed, deformed, and measured as being thinner than they actually are. Cancer tends to become more resilient to deformation compared to surrounding muscle tissue, and that effect seems limited, but in reality, one cannot rule out the possibility of it being underestimated.

In this study, the deepest part of the tumor in each image modality was visually evaluated and determined by consensus. In US, the deepest part can be evaluated in detail as an invasion front; there are reports that the risk of lymph node metastasis is high if the morphology of the invasion front is found to be irregular by comparing its morphology with that of the histopathological infiltration mode by US [41, 42]. However, it is often difficult to determine the location of the front when the contour of the deepest part is irregular. In US, the surrounding muscle tissue is hyperechoic because it is rich in fat and connective tissue, whereas, cancer is depicted in principle as hypoechoic because it has relatively little reflection, but its outer edge is accompanied by a slightly hyperechoic peripheral muscle tissue and a poorly defined region. In this study, we determined the range of the tumor, including the region of the outer margin based on our previous report [43]. Several reports have been made using blood flow imaging and elastography [44–46], but no discussion has been made regarding the location of the deepest part of the tumor.

In the UICC/AJCC 8th Edition, DOI is histopathologically defined as the vertical distance from the horizontal virtual plane (line) connecting the basement membrane of the normal mucosa adjacent to the tumor to the deepest part of the tumor. In this study, the vertical distance was from the virtual line connecting the boundary between the tumor and the normal mucosa to the deepest part of the tumor in CT and MRI, and from the virtual line connecting the basal part of the normal mucosa to the deepest part of the tumor in US in accordance with the UICC/AJCC 8th edition, in principle. However, in CT, MRI, and the histopathological specimens, for patients where a straight virtual line was considered inappropriate based on the original curvature of the tongue, a virtual curve was set with reference to the curvature of the adjacent normal mucosa and the original form of the tongue on the opposite side and measurements performed. Essentially, the virtual line is defined as a horizontal straight line, but since the tongue mucosa is arched, the measurement, as defined, may be underestimated. To date, there are also reports stating that the normal mucosal surface corresponding to the external shape of the tongue is a virtual curve in DOI measurements [2, 14, 17, 25, 47]. In addition, since the approximate shape of the tongue changes depending on the presence or absence of residual teeth, defining the virtual line may be difficult. The type of virtual line or curve that should be set must be left to the discretion of the radiologist and pathologist. However, it is essential to specify the kind of line or curve to be set in the diagnostic report. Moreover, there seems to be the need to further examine the validity of such line setting in the future. In addition, UICC/AJCC 8th edition removed invasion to the external lingual muscle from the T classification, but previous reports have indicated that the external lingual muscle appears to be relatively shallow from the normal mucosa [48]. It is reported that DOI can be judged more than 4 mm in cases of invasion to the styloglossus and hyoglossus muscles [49] and that cervical lymph node metastasis is more frequent in cases of invasion to the paralingual space [50]. In preoperative diagnostic imaging, not only DOI measurement, but also detailed evaluation of the presence or absence of invasion to the external lingual muscles is necessary.

There are several limitations to this study. First, it is a retrospective study conducted with a limited number of cases in a single institution, and the period from examination to surgery is not constant. Moreover, the changes in the tumors during that time have not been evaluated and taken into account in the analysis. It also does not take into account histopathological findings such as the degree of malignancy and infiltration mode of the cancer, which may affect the diagnosis of the deepest part of the tumor. The choice of the modality for each patient was made by the oral surgeon who was the attending physician, and there may be potential bias.

## Conclusions

With regard to DOI measurement by preoperative diagnostic imaging of T1 and T2 squamous cell carcinoma, based on the findings from the comparison with the histopathological DOI, US is the most accurate measurement method. With CT and MRI, there tends to be overestimation of about 2–3 mm.
